# Environment, dysbiosis, immunity and sex-specific susceptibility: A translational hypothesis for regressive autism pathogenesis

**DOI:** 10.1179/1476830513Y.0000000108

**Published:** 2015-05

**Authors:** Alessandra Mezzelani, Martina Landini, Francesco Facchiano, Maria Elisabetta Raggi, Laura Villa, Massimo Molteni, Barbara De Santis, Carlo Brera, Anna Maria Caroli, Luciano Milanesi, Anna Marabotti

**Affiliations:** 1 Institute for Biomedical Technologies, National Research Council, Via Fratelli Cervi 93, 20090 Segrate (MI), Italy; 2 Istituto Superiore di Sanità, Rome, Viale Regina Elena 299, 00161 Roma, Italy; 3 IRCCS “E. Medea” – Ass. “La Nostra Famiglia”, Via Don Luigi Monza, 20, 23842 Bosisio Parini (LC), Italy; 4 Dip. Scienze Biomediche e Biotecnologie, Università degli Studi di Brescia, Viale Europa 11, 25123 Brescia (BS), Italy

**Keywords:** Environmental autism, Gut dysbiosis, Immune system, Sex bias, Xenobiotics

## Abstract

**Background:**

Autism is an increasing neurodevelopmental disease that appears by 3 years of age, has genetic and/or environmental etiology, and often shows comorbid situations, such as gastrointestinal (GI) disorders. Autism has also a striking sex-bias, not fully genetically explainable.

**Objective:**

Our goal was to explain how and in which predisposing conditions some compounds can impair neurodevelopment, why this occurs in the first years of age, and, primarily, why more in males than females.

**Methods:**

We reviewed articles regarding the genetic and environmental etiology of autism and toxins effects on animal models selected from PubMed and databases about autism and toxicology.

**Discussion:**

Our hypothesis proposes that in the first year of life, the decreasing of maternal immune protection and child immune-system immaturity create an immune vulnerability to infection diseases that, especially if treated with antibiotics, could facilitate dysbiosis and GI disorders. This condition triggers a vicious circle between immune system impairment and increasing dysbiosis that leads to leaky gut and neurochemical compounds and/or neurotoxic xenobiotics production and absorption. This alteration affects the ‘gut-brain axis’ communication that connects gut with central nervous system via immune system. Thus, metabolic pathways impaired in autistic children can be affected by genetic alterations or by environment–xenobiotics interference. In addition, in animal models many xenobiotics exert their neurotoxicity in a sex-dependent manner.

**Conclusions:**

We integrate fragmented and multi-disciplinary information in a unique hypothesis and first disclose a possible environmental origin for the imbalance of male:female distribution of autism, reinforcing the idea that exogenous factors are related to the recent rise of this disease.

## Introduction

Autism spectrum disorder (ASD) is a neurodevelopmental disorder with uncertain etiology, characterized by social deficits, communication difficulties, stereotyped or repetitive behaviors, and in some cases, cognitive delays.^1^

ASD shows a striking sex bias, with a male:female ratio estimated at 3–4:1, and it usually appears by 3 years of age. Some authors have stated that ASD children often have relatively normal development during the first 12–24 months of life; then, after a period of regression, the full syndrome becomes evident.^2^

The prevalence of ASD varies among countries and it has increased dramatically in the last decades, reaching 6–7 per 1000 individuals and even more in English-speaking countries. This rise is only partially explained by changes in diagnostic criteria.^3^

Genetics is considered to play a relevant role in ASD, but only 16–17% of autistics are carriers of a known genetic variant, and only few common alterations have been recognized as candidate genes in linkage and association studies.^1^ Moreover, the gender distribution of the disease would seem to reflect a genetic X-linked pattern, but very few X-linked forms of autism susceptibility have been found to date. Therefore, in addition to genetics, possible environmental causes such as pathogens, use of antibiotics, heavy metals, chemicals, and toxins have been proposed,^4^ although in some cases their involvement has been or is still considered controversial. However, none of these factors alone can explain why only few children are sensitive to these environmental effects, why these effects occur in the first 3 years of age, and why males are more sensitive than females.

In many patients, some other diseases, such as epilepsy, metabolic defects, sleep problems, and gastrointestinal (GI) disorders are in co-morbidity with autism and, interestingly, GI disorders have a strong correlation with the severity of the disease.^5,6^

By collecting data from the literature and organizing fragmented hypotheses, we obtained a unique overview and formulated a hypothesis that tries to explain the sequence of possible causative events for regressive autism development. This hypothesis suggests how, in children not yet immune-competent, GI disorders and alterations of the intestinal ecosystem (gut dysbiosis) can establish, causing the synthesis of neuro-active molecules, leaky gut, and then neurotoxic xenobiotics (compound foreign to a living organism) production and absorption. We also found that, in animal models, some of these substances play a male-dependent neurodevelopment damage, thus reinforcing the environmental hypothesis for regressive autism. The combination of these factors, especially during infancy in which brain's sensitivity to senses and learning must be specifically timed,^7^ could lead to the development of ASD in vulnerable children. Our hypothesis can be the starting point for the prevention of all the conditions predisposing to dysbiosis and neurotoxic xenobiotics absorption, for the planning of probiotics and/or specific antimicrobial therapies, and to establish the toxin risk assessment and the daily tolerable intake.

## Discussion

### Gut and microbiota

#### The role of microbiota in human health

An increasing number of evidences point to the importance of the so-called ‘gut–brain axis’ revealing the central role of the gut microbiota (the new denomination of ‘microflora’), in the post-natal development and maturation of the immune and endocrine systems that, in turn, control the central nervous system (CNS) signaling, brain functions, and behavior.^8–10^

The gut resident commensal microorganisms consist essentially of prokaryotes, especially bacteria, and of a minor component of archaea, viruses, and eukaryotes such as fungi.

The healthy, balanced gut communities state is called ‘eubiosis’, and in adulthood is dominated by the phyla *Firmicutes* and *Bacteroidetes* followed by *Actinobacteria* and *Proteobacteria*. These microorganisms have structural and protective functions for humans, such as the intestinal wall formation, the development of both immune and endocrine systems, the increasing resistance to pathogen colonization, the detoxification and degradation of xenobiotics, the production of short-chain fatty acids (acetate, propionate, and butyrate) and vitamins, nutrient absorption, and amino acid synthesis from ammonia or urea.^11–13^ The fermentation of non-digestible carbohydrates by intestinal microbiota, leading to the production of short-chain fatty acids, has a role in several processes in the gut, such as cell proliferation and differentiation, ions and water adsorption, hormone secretion, and cytokines production (tumor necrosis factor-alpha (TNF-α), interleukin (IL)-2, IL-6, IL-10) that, in turn, activate the immune systems and regulate the leukocyte function. Moreover, by competing for the space and nutrients and by producing antimicrobial substances, commensal bacteria can effectively inhibit the growth and the proliferation of pathogens, contributing to maintain a healthy and well-balanced gut microbiota.^13,14^

Among the commensal bacteria, *Bifidobacteria* are generally considered among the most relevant beneficial bacteria and, apart from variations due to diet and age, they represent roughly the 10% of the human adult microbiota. In infants, however, a higher proportion of *Bifidobacteria* has been reported and strictly associated to an appropriate development of the immune system.^15^ Together with *Lactobacilli*, including those derived from breast milk, *Bifidobacteria* contribute to a healthy infant gut, by modulating both natural and acquired immune response.^15^ On this regard, it has been reported that *Lactobacillus salivarius* and *Lactobacillus fermentus* enhance Type 1 helper T cells cytokines production, such as IL-2 and IL-12 as well as modulate the expression of the inflammatory mediator TNF-α.^16^ More generally, it is well stated that, especially in animal models, lactic acid bacteria can enhance natural immunity by increasing the non-specific phagocytic cell function as well as cytokines, interferons, and interleukins production.^14,17^

Furthermore, human milk *Lactobacillus* strains drastically reduce (up to 46%) GI infections, as well as the general incidence rates of infections in newborns, such as those of the upper respiratory tract. Among Firmicutes, *Viridans streptococci*, and commensal staphylococci, both supplied by breast milk, help to keep under control the infection of *Staphylococcus aureus*, representing a high risk for newborns.^16,18^ In addition, studies carried on mice have been reported that lactic acid bacteria could also inhibit the GI epithelium colonization of *Candida albicans*, whose overgrowth, if not properly counteracted by commensal bacteria, can trigger a severe infection on mucosal surfaces.^19^ As by-products of fermentation, *Lactobacillus* strains produce a large amount of short-chain fatty acids, such as the butyric acid, that can effectively inhibit *C. albicans* hyphal transformation, the first step for commensal to pathogen switch, leading to hyphal invasion and the systemic infection.^19–21^

Finally, both *Bifidobacteria* and *Lactobacilli* contribute to keep an appropriate microbial homeostasis, by increasing the production of functional metabolites, such a butyrate, the main energy source of colonocytes, and by modulating the breakdown of sugars and proteins, key compounds for the intestinal function.^16^

#### Dysbiosis and GI disorders in ASD children

The development of next generation sequencing techniques has permitted to define the entire human microbioma (the genome identification of a microbiota). The pyrosequencing analysis of the 16S bacterial hyper-variable regions has discovered many species of bacteria that have never been cultured nor described previously.^22^

In this regard, several studies have been carried out in an attempt to profile the ASD microbiota, in comparison with healthy siblings and/or unrelated controls.^6,22–24^ Despite few studies reported small or no differences in the gut microbiota composition between ASD children and their unaffected siblings,^24,25^ the imbalance in the microbial gut composition in ASD samples is well stated, especially when compared with unrelated healthy controls.^6,22,23,26,27^

**Figure 1:**
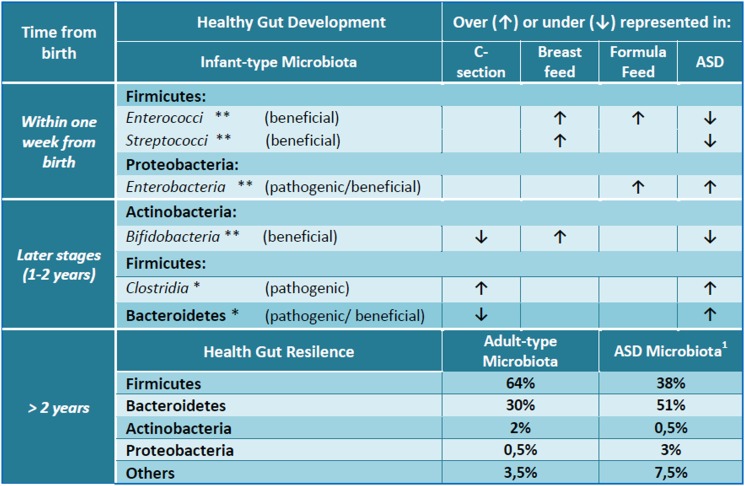
The gut microbial composition and development in healthy and autistic children. Asterisks refer to a higher (**) or low abundance (*) of microbial flora in an infant healthy gut. Arrows, instead, indicate the increase (↑) or decrease (↓) of gut microbial components according to C-section, breast or formula feed, ASD. ASD, autism spectrum disorder; C-section, cesarean section. ^1^Data by Finegold *et al.*^27^.

The meta-genomics analyses of stools from ASD patients, in fact, have revealed that ASD microbioma is significantly different from that of controls and consists of over 1000 different species in comparison with the 530 ones of healthy children.^22^ Moreover, among phyla, ASD patients have under-represented most of Firmicutes and Actinobacteria, especially *Bifidobacteria*, and over-represented most of Bacteroidetes and Proteobacteria with respect to controls (Fig. 1).^22,27,28^ Decreasing of *Bifidobacteria* that help children to develop innate immunity, in ASD microbiota, can explain pathogens overgrowth. Although Firmicutes are under-represented in their complex, the class of *Clostridia* is more abundant in ASD children with GI disorders. These bacteria are considered the most attractive responsible for the development of autism: many of them are virulent and producing toxins and antimicrobial, oxygen, and drying-resistant spores.^23,29^ Interestingly, many autistic symptoms ameliorate during oral vancomycin treatment but relapse after the agent is discontinued.^26^ Oral vancomycin is not absorbed from the intestinal wall and exerts its action exclusively on gut microbiota highlighting the crucial role of the bowel bacterial flora. Indeed, the relapse and persistence of the *Clostridia* after discontinuation of vancomycin is probably due to germination of spores,^29^ as well as the relapse of autistic symptoms is imputable to *Clostridia* re-overgrowth.

Recently, *Desulfovibrio*, a genus of Gram-negative sulfate-reducing bacteria, was found in 50% of autistic stools and in that of some siblings but not at all in controls. This sulfate-reducing and lipopolysaccharides (LPS)-producing bacterium could be responsible for the aberrant sulfur metabolism and to the high level of serum endotoxins described in ASD subjects. Despite not spore formers, *Desulfovibrio* can escape antibiotic treatments traveling, by its flagellum, within intestinal biofilm that protect it from peristalsis, antibiotics, and host defenses.^27^

Interestingly, although Bacteroidetes are over-represented in ASD stools, a significantly lower presence of genus *Prevotella*, and other fermenters has been described in the gut of ASD children with GI disorders with respect to healthy controls. These microbial differences were more strictly correlated to the severity of autistic symptoms rather than to GI disorders or specific diet regimens.^30^ *Prevotella* not only has the ability to synthesize Vitamin B1, which mitigates ASD symptoms,^31^ but it is also considered a central niche to maintain the community structure of human healthy gut microbiome.^32–34^ On the other hand, *Propionibacterium*, as well as *Clostridia* which are over-represented in ASD intestine, produces propionic acid,^35,36^ a short-chain fatty acid, able to pass the gut–blood and blood–brain barriers (BBB) altering the neurophysiological processes by binding acetyl-CoA and sequestering acetyl-carnitine involved in mitochondrial lipid transport. Recent experiments demonstrated that administration of propionic acid to young rat models caused the development of mental delay with cognitive impairments, innate neuroinflammation response, and restricted/repetitive behavioral symptoms consistent with human autism.^35^

Overgrowth of fungi, in particular of *Candida*, has also been reported as gut infection in some autistic patients,^6,37–41^ although no eukaryotic microorganism has been described in the most recent publications about autistic microbiota that had molecularly analyzed only the prokaryotic components.^5,25,40,42^ In the gut of healthy individuals, *Candida* exist only in small colonies, kept under control by the human immune system and by eubiosis. If it becomes invasive, it produces root-like structures that push through the intestinal walls increasing gut permeability and leading to the so-called leaky-gut syndrome that allows exorphins and toxins to enter the bloodstream. These molecules then trigger food intolerance and allergies. In a recent work, candidiasis and leaky-gut syndrome correlate to a subset (36.7%) of autistic patients.^43^

GI disorders affect individuals with ASD ranging widely from 9 to 70%, or higher, in different population studies, and show a strong correlation with the severity of the disease.^5,6^ GI problems of autistic children include abdominal pain, diarrhea, constipation, bloating, increased intestinal permeability, and mucosal dysbiosis.^5,42,43^ Typically, behavior symptoms and GI disturbances are manifest in parallel.^25^

**Figure 2:**
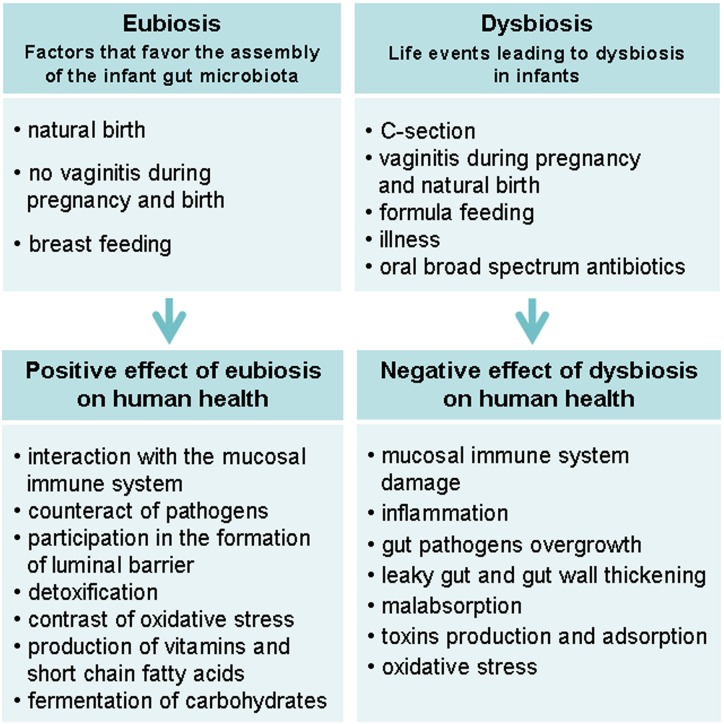
Differences between eubiosis (a healthy normal microflora) and dysbiosis establishment and consequences.

Recent findings have demonstrated that the previously described gut microbiota alterations correlate with these GI disturbances.^6,22,25^ A series of events that can occur in the very first years of life, 0–36 months, are able to alter the function and balance of gut microbiota, leading to immunological and neurological effects as we show in the following paragraphs (Fig. 2).^25,44^

#### Gut colonization

Fetal intestine is sterile until birth and its colonization, which occurs during birth, breastfeeding, and weaning, is essential for infant health. The microbial population becomes functional and steady over the time at around 3 years; before this age, infant gut microbiota is dynamic, malleable, and then relatively instable.^45^ Causes that can hamper or damage eubiosis can be cesarean section, formula-feeding, early weaning, illness, the use of oral broad-spectrum antibiotics, and malnutrition. Many of these situations not only occur in the first 3 years of life, thus disturbing the assembly of a healthy microbiota,^45^ but are also described as risk factors in ASD: (i) cesarean section (especially if scheduled) has a significantly higher frequency in mother of ASD children (27%) versus mother of healthy children (19.8%);^46^ (ii) suboptimal or absence of breast-feeding and/or early weaning may increase the occurrence of ASD;^47^ (iii) infections and the use of oral broad-spectrum antibiotics occur frequently before ASD manifestation.^4^

Cesarean section deprives the newborn of contact with maternal vaginal microbiota leading to a deficiency of strict anaerobes such *Lactobacillus* and *Prevotella* and to an increase of facultative anaerobes from mother skin, especially *Staphylococcus*, *Propionibacterium*,^48–50^ and *Clostridium* species.^48^

The gestational age of birth also influences the makeup of bowel flora, since the pattern of gut bacteria in preterm neonates differs from that of full-term newborns. Indeed, preterm newborns are hospitalized in neonatal intensive care units that routinely use formula milk and antibiotics. These practices have a negative impact on gut microbial colonization and could lead to food intolerance, to the development of necrotizing enterocolitis, and long-term neurodevelopment impairment.^13,51^ Increased prevalence of ASD in children born preterm has also been described.^52^

Furthermore, in early infancy, gut microbiota is mostly influenced by feeding: the bowel microbial composition is different between breast and formula-fed infants as well as between early and late weaning. Breast milk, containing antimicrobial molecules and prebiotic oligosaccharides, increases the beneficial component of *Bifidobacteria* and *Lactobacilli*, whereas formula milk increases the number of *Enterococci* and *Enterobacteria*.^53–56^

**Figure 3:**
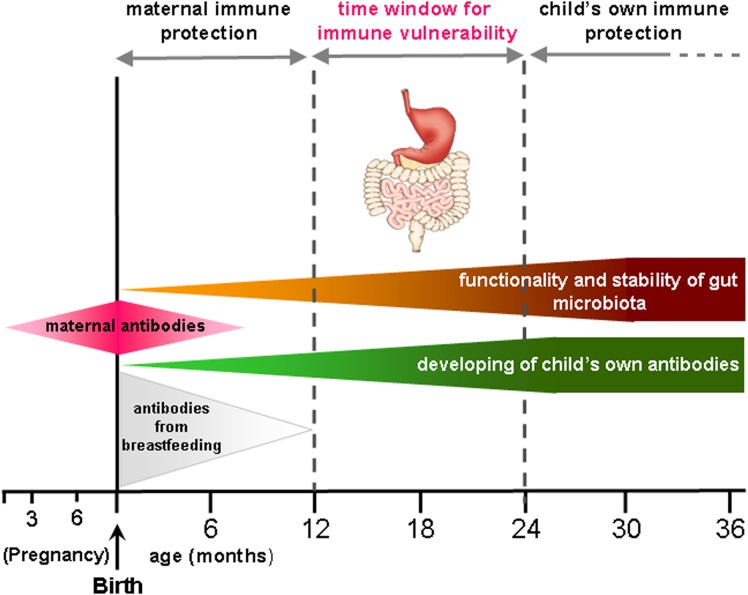
The critical window for immune vulnerability of children. At about 12 months the maternal protection is disappeared as well as antibodies from breastfeeding (especially in early weaning). On the other hand, the functionality and stability of gut microbiota as well as child's own antibodies formation are not yet reached.

On the other hand, at around 12 months, maternal immune protection decreases while the child's own immune system is not yet competent, resulting in a high susceptibility to infections^57,58^ (Fig. 3).

Many infections, especially otitis that are among the most common bacterial infections of childhood,^59,60^ are treated with oral broad-spectrum antibiotics that can perturb the gut microbiota. Moreover, many parents of autistics patients report episodes of otitis treated with oral broad-spectrum antibiotics before children manifest regressive autism symptoms, as well as some authors find ear canal malformation and/or hearing impairment more frequently in autistic children than in controls; somatic obstructions and malformation, in turn, may favor the onset of otitis and their complications.^61^ Major microbial alterations were found after antibiotic treatments that lead to a long-term decrease of particular taxa.^50,62–64^ Even more seriously, the overuse of antibiotics can lead to an increase in antibiotic-resistant pathogens^50,65^ and these phenomena have been considered as possibly related to the raise up of ASD incidence. For example, the analysis of the clinical history of 206 ASD children with no genetic alteration, birth trauma, or neurological diseases allowed to find that all of them have had recurrent bouts of otitis media treated with a mean number of 12.04 courses of clavulanic acid/amoxicillin.^66^ Although these data are lacking appropriate aged-matched controls and the author has focused her discussion on the neurotoxic effect of urea, used in drug preparation, on brain tissue, the microbiota damage caused by antibiotics themselves should also be considered. Another factor, possibly related to the recent increase of ASD, is the broad antibiotic prophylaxis against group B streptococcal infections of the newborns from the positive mothers. This clinical practice that has led to a substantial reduction in the incidence of the group B *Streptococcus* infections,^67^ it could, however, increase the risk of dysbiosis and then of gut–brain axis alteration.

On the contrary, when ASD and GI disorders occur in parallel,^25^ the use of dysbiosis-targeted antimicrobials and specific probiotics, to counteract gut pathogens overgrowth, not only ameliorates the GI disorders symptoms, but could also lead to cognitive and behavioral improvement.^10,68–73^

Moreover, almost 90% of ASD children experience feeding-related concern, especially food selectivity, with a strong preference for starches, processed food, snacks, and strong dislike for almost all vegetables and fruits and/or proteins.^47,74^ The ability of feeding to regulate the microbiota composition and growth is already well known. Recently, microbiota was found to recognize and produce neuroendocrine hormones such as γ-Aminobutyric acid (GABA) by *Lactobacillus* and *Bifidobacterium*, noradrenaline by *Escherichia, Bacillus*, and *Saccaromices,* serotonin by *Candida, Streptococcus, Escherichia*, and *Enterococcus,* dopamine by *Bacillus* and *Serratia*, and acetylcholine by *Lactobacillus*.^75^ These compounds are absorbed in the bloodstream and/or interact with the vagus nerve. These findings have lead Norris *et al.*,^76^ to propose a positive feedback loop hypothesis involving the host's food preferences and microbiota composition. Through this loop, nutrition influences gut microbiota that, in turn, produces neuroendocrine factors able to influence behavior (e.g. cognition), and food preferences.^77^ To this purpose, the gut dysbiosis of ASD children can drive their food selectivity in order to ‘feed’ pathogens and perpetuate their overgrowth.

#### Neurodevelopment

The first periods of life (0–36 months) are particularly delicate for the makeup of the gut complex community, and for immunity and neurodevelopment. Indeed, during this time, acquisition of many sensitive and cognitive processes pass through a ‘critical’ period of elevated neuron plasticity, a specific time window of neurodevelopment during which experiences trig and sculpt the neural circuits involved in that process. Distinct critical periods occur in a hierarchical sequence, starting from primary sensory acquisition up to higher cognition. For example, social relationship and communication are linked to multisensory integration involving visual, auditory, and somatosensory. So, senses (vision, hearing, touch) and intellectual abilities (language, symbols, social relationships) develop, progress, and consolidate in these times of opportunity after which learning anything new becomes very difficult or impossible: if these periods are missed by early disruption or interferences of proper senses and/or social experiences, can no longer be recovered nor senses and skills be acquired.^78,79^ In some ASD children sensory processes, such as sight and hearing or both of them, are often miswired due to somatic (ear canal malformation), functional, and infectious (otitis) causes, and the severity of sensory stimulation deprivation correlates with the severity of behavior and social relationship deficit.^60,61,80,81^ Recent findings demonstrated that critical period depends on a perfect balance of cortical excitatory and inhibitory neurotransmission regulated by inhibitor neurotransmitters GABA.^82–84^ Interestingly, dysfunction in GABA transmitter system has already been described in autism^85^ and a mistimed in the regulation of these critical periods has been proposed in the developing of ASD.^7^

#### Dysbiosis and xenobiotic production

The imbalance of microbiota composition in autistic children with GI disorders can lead to another consequence: the production of xenobiotic substances that can exert a negative systemic effect once entered the bloodstream. Indeed, a change in concentration of mammalian-microbial-cometabolites such as dimethylamine, hippurate, and phenylacetyl-glutamine has been found in the urine of ASD children.^86^

Such pathogenic linkage was also suggested by Bolte,^72^ that, starting from the evaluation of the clinical history of a child, speculated that autism could be due to a chronic tetanus infection of the intestinal tract. A possible migration, through a neural route, reaching and persistence of tetanus neurotoxin in the CNS was recently demonstrated by an experiment performed on cats: the transneuronal and transynaptic effect of the toxin injected into the medial rectus muscle of one eye causes a bilateral gaze palsy, revealing the changes in neuron connections due to the action of this neurotoxin on premotor neurons.^87^

Moreover endotoxins, especially LPS of the outer membrane of Gram-negative bacteria, induce strong inflammation, leaky gut and leaky BBB disrupting the tight junctions of the brain microvascular endothelium, thus increasing paracellular permeability.

LPS endotoxins also inhibit P-glycoprotein, a transmembrane efflux transporter able to extrude a wide range of xenobiotics out of cells, thus causing an intracellular accumulations of these substances. This protein is localized at the luminal epithelial cell surfaces of the gut, liver, proximal tubules of the kidney, hematopoietic cells, and BBB.^73,88,89^ Many diseases, including ASD, cognitive impairment, schizophrenia, Parkinson's, and Alzheimer's diseases, are associated with increased endotoxins in plasma.^89,90^

Further support for the microbial metabolite hypothesis and for the key role of the bowel bacteria in autism came from the studies on autistic children treated with antibiotics and probiotics.^91^ These evidences strongly suggest that autistic symptoms in ASD children with GI disturbances may be related to the production of neurotoxins by the gut microbiota. For example, antifungal drug therapy, such as the oral administration of nystatin, is a potential therapy in ameliorating ASD symptoms.^38^ Based on the findings of abnormal urinary metabolites, including Krebs cycle metabolites, carbohydrates, and other compounds such as furan compounds, arabinose, tartaric, and citramalic acid, of putative microbial origin in ASD children, it has been reported that antifungal treatment in patients reduces both their clinical symptoms and the abnormal urinary secretion of some chemical compounds suggesting a link between an aberrant fungal or yeast overgrowth in the GI tract and their urinary metabolite profiles.^38^ Despite any stool testing for yeast or fungi was performed in this study group and due to the small sample size, both elevated arabinose and tartaric acid could likely be associated to GI yeast overgrowth, such as *C. albicans*.^38^ Subsequent studies have confirmed that microbial metabolism results in altered metabolite profiles in children with ASD.^86,92–94^ Recently, using a ^1^H–^13^C nuclear magnetic resonance approach, it has been attempted to profile the urine metabolites in ASD, for identifying some discriminating markers between autistics and controls.^94^ Although the specificity of this new methodology needs to be validated in a larger dataset and on other developmental disorders, an increase in β-alanine, glycine, taurine, and succinate and, on the other hand, a decrease in creatinine and 3-methylhystidine have been reported in the urines of ASD children in comparison with controls.^94^ High levels of tyrosine analog 3-(3-hydroxyphenyl)-3-hydroxypropionic acid, a bacterial *m*-tyrosine metabolite that induces characteristic autistic behavioral symptoms in rats, have been found in the urine of some ASD children. Its microbial origin is supported by its decrease in urine after patients were treated for clostridial infections with metronidazole.^86,92^

Yeast and fungal infections caused by *Aspergillus* and *Candida* genus may be related to the production of the gliotoxin, a critical virulence determinant^95^ of *Aspergillus fumigatus,* that has an important immunosuppressive activity, facilitating the fungal growth and the host colonization, through the induction of a local or generalized immunosuppression.^96^ Moreover, *Candida* itself produces acetaldehyde by glucose fermentation; acetaldehyde acts on central dopaminergic system increasing dopamine levels in the nucleus accumbens, and forms ‘false neurotransmitters’ by Schiff base formation with many aminic neurotransmitters.^97^ *Candida* also affects the immune system, inducing higher productions of the interleukins IL-6 and IL-8,^98^ which were reported to be increased in the blood, brain, or cerebrospinal fluid of autistic subjects.^99^

#### Xenobiotics from food

Although there are only small-sized studies, some authors also found food protein exorphins and toxins in the fluids of ASD children.^37–39,68,86,92,100–103^ In animal models, these xenobiotics interfere with immune, oxidant, and neurological systems suggesting that these substances can interfere also with CNS.^35^

Exorphin xenobiotics, in particular, casomorphins from bovine β-casein and gliadinomorphines from wheat gluten, are opioid peptides which are formed by the degradation of food proteins, and that bind to opioid receptors inducing interferences in the dopaminergic, serotoninergic, and GABAergic pathways, thus affecting psychomotor development and emotional and motivated behavior.^100,104–106^ All these effects, known as ‘opioid peptide excess’ theory, are relevant in schizophrenia and autism and confirmed by the improvement in attention and behavior obtained in autistic patients treated with the opioid receptor blocking naltrexone.^107^ Increasing levels of exorphins also cause a fluctuating dopaminergic hyperfunction that, in animals, induces stereotypy as a typical feature.^100^

With regards to the diet, it should be pointed out that specific genetic variants of bovine β-casein are associated with higher levels of casomorphines in milk.^108^ Even if the European Food Safety Authority (EFSA) could not establish the cause–effect relationship between the oral intake of β-casomorphins and the etiology of different human diseases including autism,^109^ the potential lower production of casomorphins from milk carrying particular β-casein genetic variants or from different dairy species is an intriguing aspect which could be further taken into account in a casein-free diet planning.

Other toxic xenobiotics are mycotoxins, generated by food-contaminating microorganisms, especially fungi, that pollute the 25% of the world crop production.^110^ The toxicity of some of these mycotoxins, such as ochratoxin A (OTA), fumonisin B1 (FB1), patulin, and gliotoxin, is well known.^110–114^ Indeed, mycotoxins can increase gut permeability: OTA and patulin alter intestinal functions and intestinal barrier and transport; some experiments have demonstrated that FB1 disturbs the sphingolipid biosynthesis pathway, altering intestinal epithelial cell proliferation and leading to a damage of the barrier function.^115^ Finally, leaky gut also causes lower adsorption of nutrients, and, indeed, the deficiency of zinc, copper, and B6 vitamin was described in autistic children suffering from GI disorders.^116^

OTA, produced by *Aspergillus ochraceus*, *Aspergillus carbonarius*, and *Penicillium verrucosum*, is one of the most diffused food-contaminating mycotoxin and it has been found to be implicated in the development of human and animal neurodegenerative diseases and brain dysfunction.^113,117^

FB1, produced by *Fusarium* molds and mainly present in corn, causes neuronal tube defects in *ex vivo* mouse embryos. This effect is related to the folic acid receptor deficiency as a result of the FB1-dependent lipid rafts depletion.^114^ Among other *Fusarium* toxins, deoxynivalenol, also known as vomitoxin, is diffused in cereals and, because of its adverse effect of inducing emesis in animals, it has been pointed out as possible serotoninergic and dopaminergic receptor agonist.^118–120^

Patulin, from *Aspergillus* and *Penicillium*, that induces behavioral effects in rats,^121^ is the most common mycotoxin of apples, pears, and then of baby food such as apple and pear juice and purees,^112^ thus easily contacts the intestinal tract of infants.

Gliotoxin is a mycotoxin of the epipolythiodioxopiperazine class of fungal toxins produced by a number of *Penicillium*, *Aspergillus, Trichoderma* as well as *Gliocladium*, *Thermoascus*, and *Candida* species.^122^ Through its redox-sensitive transannular disulfide bridge, it exerts toxicity, for example, by conjugation to proteins and inactivation, by inhibiting nuclear factor kappa-light-chain enhancer of activated B-cells (NF-kB) transcription factor, and by generation of reactive oxygen species via redox cycling.^122–124^

Interestingly, several past and ongoing studies and anecdotal reports point out the positive cognitive and behavioral effects of the gluten-free-casein-free diet on ASD people, especially among children presenting GI disorders, food allergy diagnoses, and suspected food sensitivities.^125^ However, because of contradictory results, further data are needed to address limitations of current research findings.^126^ However, the efficacy of this diet could be explained by the fact that it avoids the absorption of both the most important food-derived exorphins (casomorphins and gliadinomorphines) and some mycotoxins, which often contaminate cereals and milk.

Another interesting correlation, still under debate, is between ASD and celiac disease and/or gluten intolerance.^127–129^ An intriguing correlation comes from the observation that the key molecular player in celiac disease, that is, the tissue transglutaminase enzyme^130^ has also been reported to be strongly and specifically activated by tetanus and other clostridial toxins.^131^ Furthermore, patients with ASD were found to be associated with an elevated autoantibody response to tissue transglutaminase.^132^ A further support for this correlation is the effectiveness of the gluten-free-casein-free diet, whose mechanism of action is still mostly unexplained, for children diagnosed with ASD.^125^ Finally, it is noteworthy that celiac disease, too, has recently shown a not completely explained increase of prevalence in the last decades.

These findings suggest that the clinical and molecular correlations between celiac and ASD diseases could be at least partially related to the activation of transglutaminase enzyme.

### Immune system

The neuro-immune communication, that connects immune system with CNS, is increasingly recognized. The mechanism for humoral-based neuro-immune interaction are numerous and complex and are regulated by the interface of the BBB, thus integrally belonging to the neuro-immune axis. LPS from Gram-negative bacteria and a high level of cytokines can pathologically disrupt BBB interfering with the equilibrium of the two systems.^89^

Immune aberrations, with increased levels of cytokines, have been reported in autistic children especially in those with a regressive form of the disease.^133^

Indeed, many proteins that were first discovered in the immune system were subsequently detected in the healthy, uninfected nervous system. For example, the neuronal expression profile of Major histocompatibility complex class 1 (MHC-I) proteins is dynamic during brain development and is spatially restricted throughout life, suggesting that a tight regulation may be necessary for normal neuronal functions. Neuronal MHC-I proteins are in turn modulated by increase and decrease in electrical activity and by the neuronal transcription factors CREB, NPAS4 (in inhibitory neurons), and MECP2 (in the neuron-derived cell line N2A).^134^ Interestingly, mutation and dysregulation of CREB, NPAS4, and MECP2 have been found in autism.^85,135^

Furthermore, analysis from post-mortem brains of ASD patients showed that the most consistently shared abnormality in gene expression patterns converge upon immune and enhanced oxidative stress and not upon neurodevelopmental genes, as it would be expected. Dysregulated immune responses accompanied by decreasing ATP production and/or increasing oxidative stress can potentially contribute in determining the onset and the severity of clinical symptoms, especially regression, mental retardation, and stereotypies.^136^

Recently, an *in silico* analysis of gene expression profile from ASD involved genes in the healthy developing human brain highlighted a subset of genes mainly expressed in glial cells and strictly involved in the disorder via NF-kB, TNF, and JNK network that converge on central immune-cytokine signaling pathway.^137^

Interestingly, environmental stimuli such as viruses, bacteria, or mycotoxins could interact with the inflammatory system in ASD via the induction of macrophages and the activation of NF-kB pathway.^138^ In normal conditions, NF-kB transcription factor mediates the cellular response to exogenic stressors, both by enhancing the expression of inflammatory cytokines/chemokines, and by being induced by them, as in a positive feedback loop.^139,140^ When NF-kB becomes aberrantly up-regulated, chronic or excessive inflammation is induced.^140^ An increased expression of NF-kB and an aberrant expression of cytokines, which are, in turn, up-regulated by NF-kB itself, has been found in peripheral blood samples and in neurons, astrocytes, and microglia of ASD donors,^141,142^ suggesting that ASD children may be unable to turn off the NF-kB stress-induced response.^141^

Besides modulating inflammatory system, NF-kB is also involved in cell differentiation and proliferation. As for mycotoxins, in pig kidney cell lines, FB1-induced apoptosis is due to the inhibition of protein kinase C activity, and its downstream targets, NF-kB and TNF-α. The long-term NF-kB repression leads to a consequent induction of apoptosis.^143^ Moreover, NF-kB can be also regulated by oxidative stress.^141^ OTA, in fact, can induce nitrosative stress in macrophages, brain, kidney, and liver cells, through a NF-kB-dependent induction of inducible nitric oxide synthase (iNOS), an enzyme responsible for the production of nitric oxide. This leads to DNA damage, decrease in DNA repair activity, an increase in lipid peroxidation and, potentially, an impairment in the mitochondrial activity, with a marked toxicity on the nigro-striatal dopaminergic neurons.^113,144^

Deoxynivalenol from *Fusarium* has been assessed to have effects on the transcription factors NF-kB*,* AP-1, and C/EBPβ, which have binding sites in the promoters of numerous immune- and inflammation-related gene,^145–147^ increasing the synthesis of TNF-α and IL-6 and the induction of COX-2.^148^ Interestingly, an enhanced oxidative stress in brain, as found in autistic cerebellum tissues, may affect neuronal differentiation, axonal targeting, and synapse formation.^149^

### Sex bias

Sex bias is one of the most reported detection in autism and, clearly, the identification of sex-specific pattern of gene expression can help in understanding the pathophysiology of the disorder.

Recently, Ziats and Rennert^150^ re-analyzed the sex-specific gene expression profile obtained by a transcriptomic study of normal human brain development, with a combined bioinformatics approach. They found that genes with male-specific pattern of expression are involved in the processes of immune response, cell cytoskeleton, glycoproteins/extracellular matrix, and nucleosome/chromatin that, intriguing, are also implicated in ASD. As for immune response, it was exclusively significant at the expression level, not at the genetic ones, thus suggesting that immune alteration of ASD patients could be more environmental rather than genetics. Male brain development may be naturally more susceptible to environmental adverse events than female ones, as its normal development is more strictly dependent on immune-related pathway.

At environmental level, many toxins and compounds are described to act in a sex-specific manner.

Mycotoxins can have a greater impact on males. For instance, a gender-dependent difference in the incidence of OTA-induced neural tube defects in a mouse model was described.^151^ It was speculated that these defects were due to the synergistic effect between altered BARX1 and SOX9 gene expressions. SOX9 is a transcription factor essential for skeletal development, but it is also involved in the development of the male phenotype,^152^ thus contributing to the increased risk of autism in males. Interestingly, a recent study detected up-regulation of SOX9 in autistic cases.^153^

As for FB1, it significantly affects the humoral immunity of male but not female rats^154^ and depresses the pig immune response in a sex-specific manner, with males being more susceptible than females.^155^

Genetic evidences supporting the role of sex steroids in the etiology of ASD have been already presented and explored.^156,157^ A study showed significant association between autism spectrum quotient and empathy quotient with genes related with sex steroid synthesis and transport functions (e.g. ESR2 and CYP11B1).^157^ More recently, a new candidate gene for autism, retinoic acid-related orphan receptor-alpha, a hormone-dependent receptor factor, has been introduced: its expression can be regulated by male and female hormones through their respective receptors, and one of its transcriptional targets is CYP19A1 (aromatase), an enzyme responsible for the conversion of testosterone to estrogen.^158^ In this context, the xenobiotics may interfere on critical functions: zearalenone, for example, a *Fusarium* toxin commonly found in maize, is a potent estrogen-like toxicant acting as an estrogen agonist in the brain.^159^

Concerning acetaldehyde, produced by *Candida,* a study about alcoholism described sex differences in peak acetaldehyde (the first metabolite of ethyl alcohol) concentration, with males showing a higher value than females.^160^

Very recently, the key role of microbiota on gut–brain axis was investigated and demonstrated using germ-free (GF) mice. Indeed these studies showed that, during early life, GF mice, especially males, manifest significant repetitive behaviors, social avoidance, and deficit that resemble those described in patients with neurodevelopment disorders such as autism. At biochemical level, GF mice presented altered monoamine neurotransmitter levels in the brain, imbalanced neurotrophin levels in the hippocampus and amygdala, increased serotonin level in the hippocampus, and increased neuroendocrine response to stress.^9,161^ In order to reverse the social deficit, a post-weaning colonization of the gut of GF male mice was carried out and the social behavior was evaluated and compared with that of conventionally colonized mice. The results revealed that GF colonized mice regain the social motivations and interests for social novelty and regulate repetitive behaviors, thus underline the key role of microbiota in neurodevelopment and social behaviors. As these aspects are relevant in autism, that also has a similar male prevalence, they should be important clues for understanding the role of microbiota in the pathogenesis of the disorder and for planning novel therapies for microbial modifications,^161^ such as probiotics and dietary interventions.

Also, sex-based differences in immunity are known, since human illnesses affect males and females differently. In general, both the proportion of individuals are infected, and the severity of infection are higher in males than females for viral, bacterial, fungal, and parasitic diseases.^162,163^

Regarding the opioid peptides casomorphins, sex differences in opiate sensitivity have been demonstrated in multiple pre-clinical studies using pain models, and morphine resulted less potent in women compared with men. This is most likely due to differences in opiate receptor density, binding, and localization, as well as sex differences in the anatomy and physiology of opiate-responsive neural circuits.^164–166^ In animal models, the expression of μ-opioid receptors in the ventro-lateral periaqueductal gray is sexually dimorphic and males have significantly higher levels of μ-opioid receptors compared with females.^167^

### Critical analysis of literature

We have constructed this translational hypothesis for regressive autism pathogenesis assembling, in a complete and logical sequence, many articles and reviews containing different analyses and partial hypotheses for ASD etiology, spanning from genetics to environmental evaluations so as from clinical to pre-clinical studies.

Regarding genetics studies, many and consistent improvements in gene alteration analyses and discovery have been done since the newest technologies have been developed. Indeed, genome wide association has been applied to a great number of patients, thus allowing to find a robust correlation between genetic markers and specific clinical aspects of the disease. On the other hand, the development of next generation sequencing technologies has allowed to perform *de novo* sequencing, revealing new genes and new alterations causative of ASD. Unfortunately, the power and possibilities offered by these technologies in transcriptome analyses are, in ASD studies, limited to RNA from lymphoblastoid cells, as samples from CNS are, obviously, very rarely available.^136,137^ However, in spite of the great progress in genetics analyses, only ∼17% of patients carry a genetic alteration unequivocally linked to ASD and very few of them can explain the male excess or the regression to autism that sometimes happen in healthy children between 12 and 36 months.

Many interesting articles about environmental causes of ASD have been published but none explained exhaustively why these agents can impair only few children, which are the predisposing conditions, why some healthy children progressively or suddenly regress to autism and why more males than females are involved. Moreover, some of them are contradictory and this may be due to the sized set and kind of samples analyzed: different age and clinical evaluations and, obviously, an over-representation of males (Table 1).

**Table 1:** Review analysis of literature regarding the most relevant environmental factors or stressors, associated to ASD

Environmental factors/stressors	Relevant literature	Study type	Association with:		Main findings	Comments
			ASD	ASD + GI disorders		
Antibiotics	Sandler *et al.*^69^	Clinical	Yes	Yes	Short-term improvement after low doses of vancomycin and probiotic therapy in a sub-group of regressive ASD children	Small sample size. The reported improvements waned at follow-up. Further investigations are required
	Atladóttir *et al.*^59^	Clinical	No	–	Population-based cohort study reporting no associations for ASD and mild infections, febrile episodes, or the use of antibiotics during pregnancy	Methodological limitations due to self-reported data/incomplete information
Diet and food xenobiotics	Batista *et al.*^129^	Clinical	No	No	No relevant associations among ASD, celiac disease, and glutein sensitivity	Poor diagnosis for ASD Because of the age of celiac disorder's onset is variable, it is possible that ASD patients would still develop this disease in the future
	Pennesi and Klein^125^	Clinical	Yes	Yes	Effectiveness of glutein/casein free diet in a sub-group of ASD children	Retrospective study Potential diet errors
	Reichelt *et al.*^106^	Clinical	Yes	–	Finding of exorphins in urines of ASD children	Further studies are required to strengthen the role of bio-active peptides in ASD
	Critchfield *et al.*^71^	Review	–	–	By comparing GI disorders in ASD and IBD, the supply of probiotics for ameliorating ASD symptoms is strongly encouraged	Need for pre-clinical/clinical trials
	Williams *et al.*^42^	Clinical	Yes	Yes	Insights between human gene expression and gut bacterial community composition.	Small sample size.
	Genuis *et al.*^128^	Clinical	–	Yes	Glutein-free diet effectiveness in a 5-years-old child with ASD and suspected celiac disease	Case report
	Cass *et al.*^126^	Clinical	No	–	Lack of evidence for opioid peptides in urine of male ASD children. No effectiveness for opioids in predicting or monitoring the effects of casein/glutein-free diet	No clinical evaluation for GI disorders in the samples. Further studies are required to validate the effectiveness of casein/glutein-free diet in ASD
Microbial metabolites/end-products	Frye and MacFabe^36^	Clinical	Yes	–	Short-chain fatty acids and acetyl-carnitine abnormalities in ASD patients versus controls	Further studies are required to strength the similarity between animal models and ASD and to evaluate the role of microbiota as potential PPA source
	Mavel *et al.*^94^	Clinical	Yes	–	Aberrant urine metabolic profiling in ASD children versus controls by ^1^H^13^C NMR	Needs for larger sample size Needs for testing the methodological specificity
	Kuwabara *et al.*^90^	Clinical	Yes	–	CE-TOFMS revealed aberrant metabolites, associated with oxidative stress and mitochondrial dysfunction, in the plasma of ASD adult males	Only adult males taking into account Needs for testing the methodological specificity and sensitivity
	Ming *et al.*^93^	Clinical	Yes	Yes	Altered gut microbial metabolites in urines of ASD children, with a stronger association in patients with GI disorders, suggesting a link between gut-dysbiosis and metabolic perturbances	No differences between ASD patients and controls have been found, by considering gender, diet, and vitamins supplementations. No evaluation for microbial gut-composition in ASD versus control children
	Kalużna-Czaplińska and Blaszczyk^39^	Clinical	–	Yes	High levels of arabinose found in autistic children and positive effects of probiotics in reducing them	No controls evaluated. A possible involvement of *Candida* is suggested Preliminary study
	MacFabe *et al.*^35^	Pre-clinical	Yes	–	Effects of propionic acid (PPA) in inducing autistic-like behaviors in male adolescent rats	These findings support further evidences on the effects of PPA in young rodents. Further works are required to validate this model in clinical studies
	Shaw^92^	Clinical	Yes	–	Higher concentrations of HPHPA in the urine of ASD children, in comparison to aged-matched controls, and in one adult affected by *C. difficile* infection	No clinical evaluation for GI disorders in the samples. No analysis on the ASD stool samples
	Yap *et al.*, 2010^102^	Clinical	Yes	–	Urinary metabolic alterations in ASD children versus siblings and controls identified by ^1^H NMR, and potentially associated with gut disorders	No evaluation for gut microbiota in ASD samples. Needs for larger sample of early onset patients to clarify the role of these metabolic differences in the autism etiology
	Shaw *et al.*^38^	Clinical	Yes	–	Positive effect of the antifungal therapy in reducing fungal metabolites in ASD patients	No data reported about GI disorders or the analysis of stool samples
Gut microbiota	Gondalia *et al.*^24^	Clinical	No	No	Pyrosequencing analysis on ASD patients with and without GI disorders and siblings. No evidences for the involvement of gut dysbiosis in ASD	No evaluation for eukaryotic gut microbiota, viruses, or protozoa as well as for dietary differences among participants. Microbial end-products/metabolites should also be studied in the future
	Adams *et al.*^6^	Clinical	–	Yes	Strong correlation with GI disorders and autism severity; lower levels of *Bifidobacteria* have been found in ASD patients versus healthy controls	No differences for yeasts was reported. Cultural and biochemical tests for microbiota profiling
	Finegold *et al.*^22^	Clinical	–	Yes	Positive correlation with the highest levels of *Bacteroide*s in ASD children and autism severity. Significantly differences have been also found for *Actinobacterium* and *Proteobacterium*	No differences between ASD patients and healthy siblings. No evaluation for the potential diet effects on gut microbiota
	Parracho *et al.*^25^	Clinical	Yes	Yes	Association between the high levels of *Clostridium* spp*.* and GI disorders in ASD patients versus healthy, unrelated, controls	No differences found between ASD patients and healthy siblings
	Song *et al.*, 2004^23^	Clinical-methodol	Yes	–	Identification with Real-Time PCR of *Clostridiales* spp. in ASD stools	Small sample size. A comparison group of ASD patients with GI disorders was not included
	Finegold *et al.*^40^	Clinical	–	Yes	Higher levels of *Clostridium* spp. in ASD feces versus healthy controls	Analysis of both fecal flora and gastric and small-bowel specimens. Small sample size

ASD, autism spectrum disorder; GI, gastrointestinal; IBD, intestinal bowel disease; PPA, propionic acid; HPHPA, 3-(3-hydroxyphenyl)-3-hydroxypropionic acid; NMR, nuclear magnetic resonance spectroscopy; CE-TOF-MS, capillary electrophoresis time-of-flight mass spectrometry.

Indeed, among the many possible and controversial environmental causes, we focused on the most emerging and convincing evidences and hypothesis: the involvement of gut dysbiosis in the etiology of the disease. Once supported only by poorly powered studies, describing very few cases, this hypothesis is now further supported by subsequent microbioma analyses: indeed, recent next generation sequencing meta-analyses^22,91^ of stools from autistic patients revealed a microbioma imbalance that might explain the immune system alteration already described in ASD children, although the role of fungi is not taken into account.

On the other hand, the most studied and concordant literature refers to the involvement of immune system in ASD etiology: the involvement of cytokines and other pro-inflammatory compounds in ASD is already well-known.^4,99,133^

Referring to sex disparity, one of the most typical issues in ASD, neither environmental studies nor those about molecular causes already published try to investigate this sex imbalance, making this issue still incomplete and unexplained. Therefore, in this regard, the studies about this topic are still lacking, making difficult to carry out a comprehensive critical analysis. However, we tried to further analyze how gene expression, immunological susceptibilities, and environmental stressors, such as xenobiotics, could affect mainly males than females, highlighting the role of environment, rather than genetics, in male-predisposing to ASD. Taking all these findings together, our hypothesis organizes partial and multidisciplinary scientific data and hypotheses in a timing sequence and in a logic way. It tries to explain what naturally happens in the first 3 years of life regarding the development and interaction of immune, gut-microbial, and nervous systems and what sequence of events can interfere with this harmonization causing regressive autism especially in males.

## Conclusions

**Figure 4:**
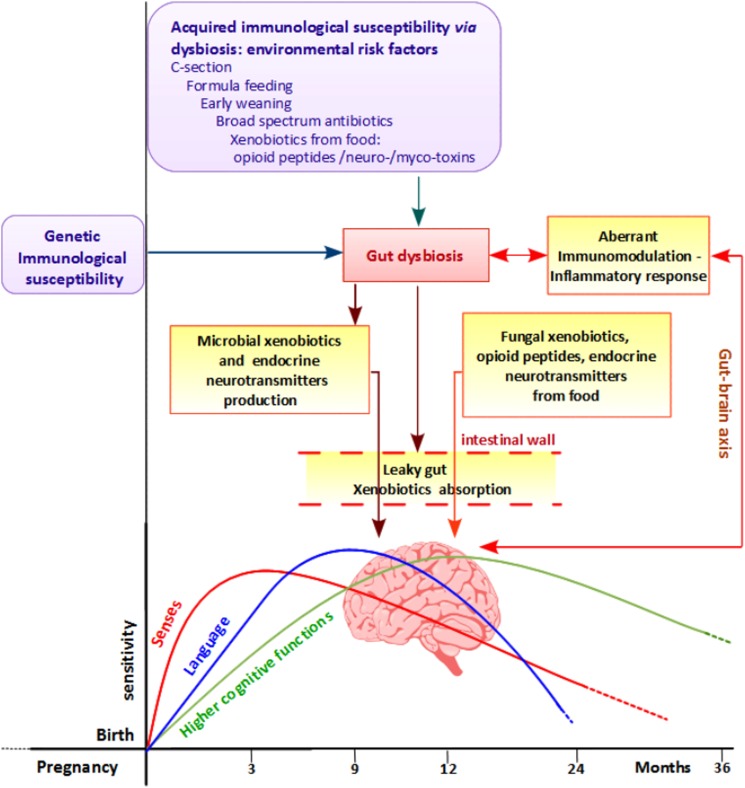
Suggested pathogenesis for autism. Genetic/immunological susceptibility and environmental risk factors could enhance gut dysbiosis, leading to an aberrant inflammatory response, to an abnormal production of microbial end-products, and to leaky gut. The latter can enhance mal-absorption of both microbial and exogenous xenobiotics derived from diet. Once absorbed in the bloodstream, all these compounds can affect the normal brain development and function both directly and impairing the immune system: the latter creates a loop, of aberrant gut–brain axis communication that contributes to enhance these aberrant physiological responses. Finally, endogenous or exogenous stressors might have an impact in the development of senses, language, and higher cognitive functions developing and integrating in the first period of life.

During the first 3 years of life, infant health is particularly vulnerable: at about 1 year the maternal immune protection decreases while the child's own immune system is not yet completely competent.^57^ Also, the gut microbiota that protects against pathogens and interacts with immune system too is not entirely stable and working until 3 years. This creates a time window of particular vulnerability to infections that, especially if treated with broad-spectrum antibiotics, favors gut dysbiosis and GI disorders. In addition, the published reports highlighted the importance of antibiotic use/overuse and onset of ASD, indicating a 67% of probability of co-occurrence.^72^ In our opinion, dysbiosis creates a vicious circle leading to a further impairment of the immune system, to the production of microbiota toxins and neurochemical compounds, to the reduction of detoxification, and to the leaky gut causing the adsorption of many xenobiotics (Fig. 4). Some of them promote redox imbalance, intestinal permeability, immunosuppressant, and a sex-specific neurotoxicity, leading to the development of regressive autism, especially in males. This gender-related xenobiotics affect couples with a well-known intrinsic male vulnerability of the immune system.

In conclusion: (i) GI disorders should be an expression of an altered intestinal barrier depending on dysbiosis. This could lead to toxins production and adsorption, interfering with normal neurodevelopment in vulnerable children, causing their regression to autism; (ii) the metabolic pathways, altered in ASD children, could be affected by genetic defects or dysregulated by xenobiotics interference; (iii) some enterotoxins and food-xenobiotics promote a male-specific neurotoxicity, thus reinforcing a possible environmental origin for the ASD male excess and the recent rise of the disease prevalence; (iv) the genetic predisposition to microbiota effects and sensitivity to xenobiotics toxicity in males should be investigated, as well as the possible additive and/or synergistic effects of toxins on general population should be defined to establish the risk assessment and the daily tolerable intake.
